# 633. Preliminary Results from a Phase 1 Single Ascending-Dose Study Assessing Safety, Serum Viral Neutralizing Antibody Titers (sVNA), and Pharmacokinetic (PK) Profile of ADG20: an Extended Half-Life Monoclonal Antibody Being Developed for the Treatment and Prevention of Coronavirus Disease (COVID-19)

**DOI:** 10.1093/ofid/ofab466.831

**Published:** 2021-12-04

**Authors:** Helen Paguntalan, Zoltán Magyarics, Lynn E Connolly, Ellie Hershberger, Kristin Narayan, Deepali Gupta, Paul G Ambrose, Frank Engler, Ed Campanaro, Anita F Das, Pete Schmidt

**Affiliations:** 1 WCCT Global, Cypress, California; 2 Adagio Therapeutics, Inc., Waltham, Massachusetts; 3 Certara, Buffalo, New York

## Abstract

**Background:**

ADG20 is a fully human IgG1 monoclonal antibody engineered to have high potency and broad neutralization against severe acute respiratory syndrome coronavirus 2 (SARS-CoV-2) and other SARS-like CoVs with pandemic potential by binding to a highly conserved epitope in the receptor-binding domain (RBD) of the spike protein. The Fc region of ADG20 has been modified to provide an extended half-life. ADG20 is in clinical development for the treatment and prevention of COVID-19.

**Methods:**

This is an ongoing Phase 1, randomized, placebo (PBO)-controlled, single ascending-dose study of ADG20 administered intramuscularly (IM) or intravenously (IV) to healthy adults aged 18–50 years with no evidence of prior or current SARS-CoV-2 infection. Participants were randomized 8:2 in 3 cohorts (N=10/cohort: n=8 ADG20, n=2 PBO): ADG20 300 mg IM, 500 mg IV, and 600 mg IM. Safety, tolerability, PK, and sVNA titers were assessed up to 3 months post dose. Serum ADG20 concentrations were measured with a validated hybrid ligand binding liquid chromatography–mass spectrometry (MS)/MS assay. sVNA titers against authentic SARS-CoV-2 were determined by a plaque reduction neutralization assay.

**Results:**

Overall, 30 participants received ADG20 (n=24) or PBO (n=6). Blinded safety data for all cohorts and PK/sVNA titer data for the 300 mg IM cohort are reported. Through a minimum of 10 weeks post dose, no study drug-related adverse events (AEs), serious AEs, injection site reactions, or hypersensitivity reactions were reported. The observed preliminary PK profile was dose proportional, consistent with an extended half-life monoclonal antibody, and well predicted by translational physiologically-based PK modeling. The measured 50% sVNA titer (MN50; geometric mean [coefficient of variation, %]) was 1382 (32.7%) 13 days after a single 300 mg IM dose. These values are within the range of peak serum neutralizing antibody titers reported for COVID-19 mRNA vaccines.

**Conclusion:**

A single dose of ADG20, up to 600 mg IM, was well tolerated. Preliminary PK and sVNA titer profiles support the ongoing Phase 2/3 trials of ADG20 at a 300 mg IM dose for the prevention of COVID-19 (EVADE: NCT04859517) and treatment of ambulatory patients with mild to moderate COVID-19 (STAMP: NCT04805671).

**Disclosures:**

**Helen Paguntalan, MD**, **Adagio Therapeutics, Inc.** (Scientific Research Study Investigator) **Zoltán Magyarics, MD, PhD**, **Adagio Therapeutics, Inc.** (Consultant) **Lynn E. Connolly, MD, PhD**, **Adagio Therapeutics, Inc.** (Employee) **Ellie Hershberger, PharmD**, **Adagio Therapeutics, Inc.** (Employee) **Kristin Narayan, PhD**, **Adagio Therapeutics, Inc.** (Employee) **Deepali Gupta, BS**, **Adagio Therapeutics, Inc.** (Employee) **Paul G. Ambrose, PharmD**, **Adagio Therapeutics, Inc.** (Employee) **Frank Engler, PhD**, **Adagio Therapeutics, Inc.** (Independent Contractor) **Ed Campanaro, BSN, MSHS**, **Adagio Therapeutics, Inc.** (Employee) **Anita F. Das, PhD**, **Adagio Therapeutics, Inc.** (Consultant) **Pete Schmidt, MD**, **Adagio Therapeutics, Inc.** (Employee)

